# Cost-effectiveness analysis of branded and authorized generic celecoxib for patients with chronic pain in Japan

**DOI:** 10.1371/journal.pone.0253547

**Published:** 2021-07-06

**Authors:** Yusuke Karasawa, Isao Kamae, Kazutaka Nozawa, Shigeki Zeniya, Tatsunori Murata, Satoshi Soen, Choitsu Sakamoto

**Affiliations:** 1 Medical Affairs, Viatris Pharmaceuticals Japan Inc., Tokyo, Japan; 2 Department of Health Policy and Technology Assessment, Graduate School of Public Policy, The University of Tokyo, Tokyo, Japan; 3 CRECON Medical Assessment Inc., Tokyo, Japan; 4 Soen Orthopaedics, Osteoporosis and Rheumatology Clinic, Kobe, Japan; 5 Department of Gastroenterology, Graduate School of Medicine, Nippon Medical School, Tokyo, Japan; University of South Carolina College of Pharmacy, UNITED STATES

## Abstract

**Objectives:**

The aim of this study was to examine the cost-effectiveness of branded and authorized generic (AG) celecoxib for chronic pain patients with osteoarthritis (OA), rheumatoid arthritis (RA), and low back pain (LBP), using real-world cost information for loxoprofen and pharmacotherapy for gastrointestinal bleeding.

**Methods:**

This cost-effectiveness analysis was performed as a long-term simulation using the Markov model from the Japanese public healthcare payer’s perspective. The analysis was conducted using loxoprofen with real-world weighted price by branded/generic distribution (hereinafter, loxoprofen with weighted price) as a comparator. In the model, we simulated the prognosis of patients with chronic pain by OA, RA, and LBP treated with loxoprofen or celecoxib, over a lifetime period.

**Results:**

A cost-increase of 129,688 JPY (1,245.00 USD) for branded celecoxib and a cost-reduction of 6,268 JPY (60.17 USD) for AG celecoxib were recognized per patient in lifetime horizon, compared to loxoprofen with weighted price. No case was recognized to reverse the results of cost-saving by AG celecoxib in one-way sensitivity analysis. The incremental cost-effectiveness ratio of branded celecoxib attained 5,403,667 JPY/QALY (51,875.20 USD/QALY), compared to loxoprofen with the weighted price.

**Conclusion:**

The current cost-effectiveness analysis for AG celecoxib revealed its good value for costs, considering the patients’ future risk of gastrointestinal injury; also, the impact on costs due to AG celecoxib against loxoprofen will be small. It implies that the disadvantage of AG celecoxib being slightly more expensive than generic loxoprofen could be offset by the good cost-effectiveness during the prognosis.

## Introduction

There are a lot of patients suffering from chronic pain due to underlying diseases such as osteoarthritis (OA), rheumatoid arthritis (RA), and low back pain (LBP) in Japan. The prevalence of those diseases was reported as 42.0% (male knee OA), 61.5% (female knee OA), 15.7% (hip OA), 1.0% (RA) and 10.3% (LBP), respectively [1–4]. In terms of disability-adjusted life years (DALYs), “back and neck pain” is globally ranked as the 3rd highest cause, and 10th and the 54th for OA and RA, respectively [5].

Nonsteroidal anti-inflammatory drugs (NSAIDs) exert analgesic and anti-inflammatory effects by inhibiting cyclooxygenase (COX). COX represents a family of isoenzymes that includes inducible COX-2, which is transiently produced and involved in inflammation and pain, and COX-1, which is constantly present and involved in gastrointestinal mucosal protection and platelet aggregation. Traditional nonselective NSAIDs block both types of COX enzymes; they are considered to cause side effects such as gastrointestinal injury as a result of COX-1 inhibition [6]. In a study of patients receiving NSAIDs for more than 28 days in Japan, 62.8% of patients had some reported abnormality in the upper gastrointestinal tract, of which 10.3% had ulcer [7]. Gastrointestinal injury caused by NSAIDs can be improved by discontinuing administration of the drug; however, discontinuation can make it impossible to manage pain due to underlying diseases such as osteoarthritis (OA), rheumatoid arthritis (RA), and low back pain (LBP). Some cases of serious gastrointestinal complications (perforation, obstruction, bleeding, ulcer) can lead to hospitalization and hemostatic surgery, and in the worst case, death. Accordingly, prevention of gastrointestinal injury due to NSAIDs is important not only in terms of patient prognosis but also in terms of health economics.

Celecoxib, a selective inhibitor of COX-2, has the same anti-inflammatory and analgesic effects as conventional non-selective NSAIDs, but it has a low affinity for COX-1. Therefore, side effects such as gastrointestinal injury occur less frequently with celecoxib than with non-selective NSAIDs such as loxoprofen sodium (hereinafter, loxoprofen), ibuprofen or diclofenac. Accordingly, fewer side effects are expected when celecoxib is used for pain management, which should improve patient quality of life (QOL) and reduce medical costs.

A review article examining several clinical trials of celecoxib as part of conventional treatment in Japan [8] showed that loxoprofen-associated symptomatic ulcers and gastrointestinal bleeding occurred in about 0.7% of Japanese patients, whereas in patients treated with celecoxib, the incidence was 0.1%. Kawaguchi et al. showed that branded celecoxib was cost-effective with an incremental cost-effectiveness ratio of 3 million JPY and 5 million JPY (28,800 USD and 48,000 USD) compared with branded and generic loxoprofen, respectively [9].

Authorized generic (AG) celecoxib was launched by Pfizer Japan Inc. in 2020. AGs are authorized versions of patented drugs marketed by brand pharmaceutical companies at generic prices without brand names; their constitution is essentially identical to the branded version. The daily price of branded celecoxib was 138 JPY (1.31 USD, 1 JPY = 0.0096 USD) as a dose of 200mg. The daily price of AG celecoxib at the same dose was 39.2 JPY (0.38 USD). Such cost benefits potentially improve patient access to pain management with celecoxib. However, the price of AG celecoxib is still slightly higher than that of generic loxoprofen, which is universally used for the pain relief in Japan. The cost-effectiveness of AG celecoxib compared with loxoprofen has not yet been demonstrated. The cost-effectiveness analysis of branded celecoxib by Kawaguchi et al. was based on separate analyses against branded or generic loxoprofen only, not based on real-world price distribution [9]; it would therefore be meaningful to update the analysis because new pharmacotherapeutic options for prevention and treatment of gastrointestinal bleeding have since become available.

The aim of this study was to examine the cost-effectiveness of branded and AG celecoxib for chronic pain patients with OA, RA, and LBP, using real-world cost information for loxoprofen and pharmacotherapy for gastrointestinal bleeding.

## Methods

### Study design

This cost-effectiveness analysis was performed as a long-term simulation using the Markov model. The analysis was conducted using loxoprofen with real-world weighted price by branded/generic distribution (hereinafter, loxoprofen with weighted price) as a comparator, which is the most widely used NSAID in Japan [10]. In the model, we simulated the prognosis of patients with chronic pain by OA, RA, and LBP treated with loxoprofen or celecoxib, over a lifetime period. The cost-effectiveness of celecoxib was evaluated by incremental cost-effectiveness ratio (ICER), which represents an incremental cost per quality-adjusted life year (QALY) gained against comparator and considered whether the ICER values were lower than the reference value or not. From the Japanese public healthcare payer’s perspective, only direct medical costs were included and the discount rate for costs and effectiveness was 2% per year [11]. No inflation/deflation rate was applied for cost calculation because the recent inflation/deflation rate in Japan is nearly zero and the adjustments for them are not described in the Japanese guideline for cost-effectiveness evaluation [11]. The methods of the study followed the Consolidated Health Economic Evaluation Reporting Standards (CHEERS) Statement [12].

### Model structure

**Fig 1:**
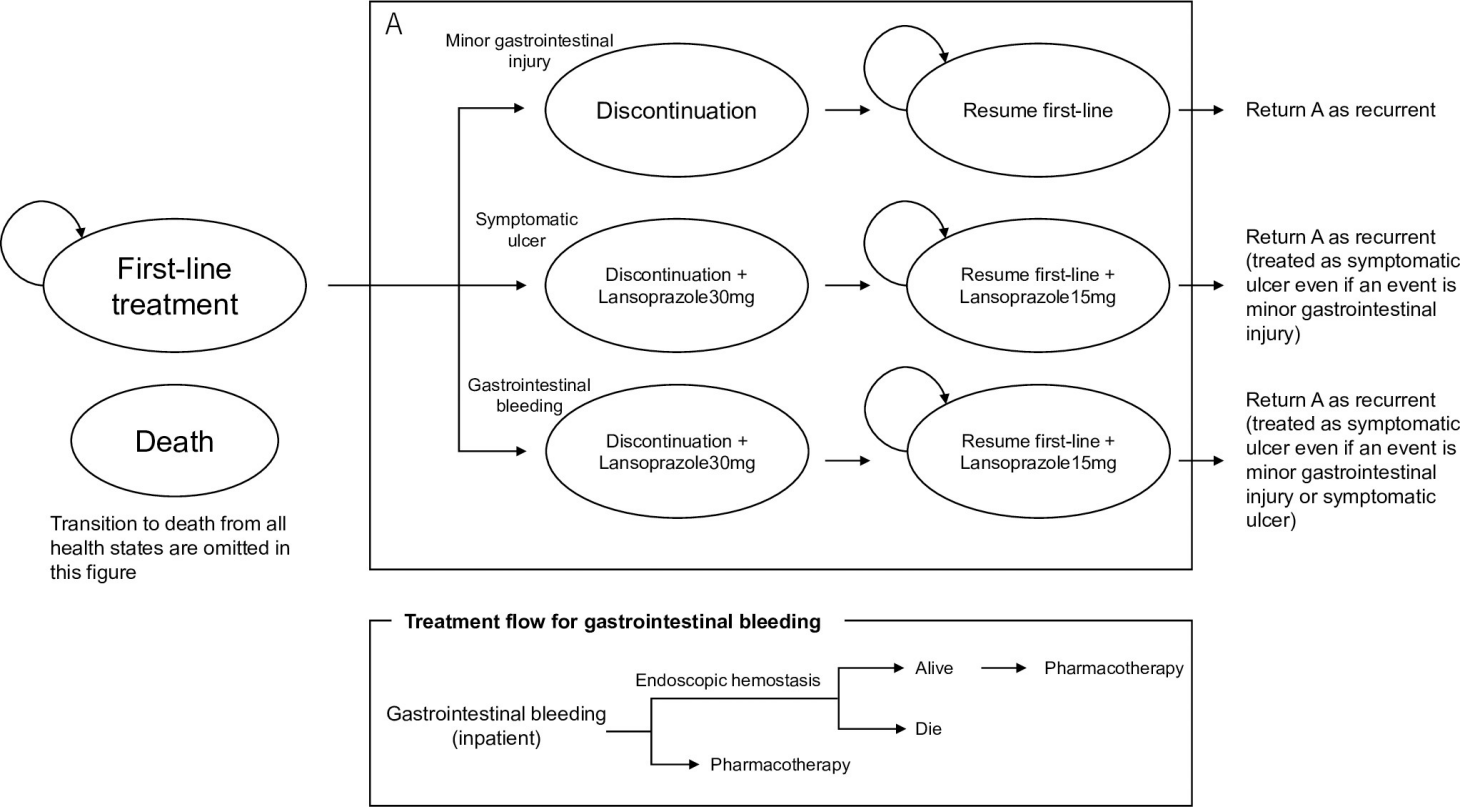
Model structure.

The target patients for this analysis were similar to those who participated in three clinical trials comparing celecoxib versus loxoprofen in Japan [6–8]. This model not only included symptomatic ulcer and gastrointestinal bleeding but also subsequent minor gastrointestinal injury as an event related to NSAIDs, by using the results of the three clinical trials [13–15] (Fig 1).

The effect of celecoxib on each event was determined in terms of the relative risk (RR) versus loxoprofen. In addition, the effect of increasing the risk of each event due to aging was considered [16,17].

The reference age for each event risk was 57 years, which was the weighted average of the three average ages in the three clinical trials [13–15].

### Clinical parameters

The clinical parameters in the model were listed in Table 1.

**Table 1:** Clinical parameters.

Parameters		Setting		Source
		Loxoprofen 180mg	Celecoxib 200mg	
**Gastrointestinal injury risk/3 months**	Minor gastrointestinal injury*1	11.3%	11.3% x 0.982	Abe et al. (2006) [13]: Sugawara (2006) [14]: Kikuchi et al. (2009) [15]*2
	Symptomatic ulcer	0.42%	0.42% x 0.201	Sakamoto and Soen (2011) [8]
	Gastrointestinal bleeding	0.25%	0.25% x 0.000	Sakamoto and Soen (2011) [8]
**Age-specific RR of symptomatic ulcer risk (also applies to minor gastrointestinal injury)**	49 years or younger	0.84		Sugano et al. (2012) [16]
	50–59 years	1 (standard)		
	60–69 years	1.19		
	70–79 years	1.41		
	80 years or older	1.68		
**Age-specific RR of gastrointestinal bleeding**	49 or younger	0.56		Hernández and Rodriguez (2000) [17]
	50–59 years	1 (standard)		
	60–69 years	1.33		
	70–79 years	2.50		
	80 years or older	5.11		
**Implementation rate of endoscopic hemostasis in gastrointestinal bleeding**		20%		Hiraishi (2010) [18]*3
**Mortality in cases of endoscopic hemostasis**		69 years old or younger: 0.42% 70 years old or older: 1.00%		Higa et al. (2011) [19]
**Recurrence rate of minor gastrointestinal injury/6 months**		22.3%	22.3% x 0.982	Yeomans et al. (1998) [20]
**Recurrence rate of symptomatic ulcer/6 months**		8.2%	8.2% x 0.201	Sugano et al. (2012) [16]
**Recurrence rate of gastrointestinal bleeding/6 months**		1.1%	1.1% x 0.000	Sugano et al. (2012) [16]

RR: Risk ratio.

The effect of increasing event risk at each age was set as the RR to the event risk at the reference age. The risk-increasing effect by age of minor gastrointestinal injury was assumed to be equivalent to the risk-increasing effect by age of symptomatic ulcers.

Regardless of which event occurred, NSAIDs were washed out for 3 months. In the event of symptomatic ulcer and gastrointestinal bleeding, the therapeutic administration of proton-pump inhibitors or proton-pump inhibitor/potassium-competitive acid blocker was anticipated. If minor gastrointestinal injury and symptomatic ulcer occurred, outpatient management was required. If gastrointestinal bleeding occurred, the patient was subject to hospitalization. Hiraishi (2010) commented: “Clinically significant bleeding occurs in 20% of patients with ulcer bleeding, and in about 80% of the patients bleedings stop spontaneously and usually recover without causing any problems [18].” It was therefore assumed that endoscopic hemostasis would be performed in 20% of gastrointestinal bleedings, and death (death due to ulcer) was considered in the cases of endoscopic hemostasis.

After the occurrence of each event (second-line treatment), the following actions were taken: In the case of minor gastrointestinal injury, the NSAIDs (first-line treatment: loxoprofen or celecoxib) were restarted and no new treatment was added (if subsequent recurrence was symptomatic ulcer or gastrointestinal bleeding, the treatment of each applicable event was followed); in the case of symptomatic ulcer and gastrointestinal bleeding, prophylactic administration of proton-pump inhibitors or proton-pump inhibitors/potassium-competitive acid blocker was started to prevent recurrence, in addition to resumption of NSAIDs (first-line treatment). In actual practice, patients with repeated gastrointestinal tract injuries on multiple occasions were discontinued from treatment with NSAIDs. Thus, in this analysis, the number of occurrences of each event (symptomatic ulcer and gastrointestinal bleeding) during lifetime was assumed as up to two (the event would not occur thereafter), and NSAIDs were discontinued after the second event occurred.

### Cost parameters

The cost parameters in the model were listed in Table 2.

**Table 2:** Cost parameters.

Parameters	Setting	Source
**Loxoprofen 180mg/day/month**	927 JPY (8.90 USD)	60mg x 3/day = 30.9 JPY (0.30 USD) (weighted by branded/generic ratio in the national statistics 2017)
**Branded celecoxib 100mg x 2/day/month**	4,140 JPY (39.74 USD)	100mg x 2/day = 138 JPY (1.32 USD)
**AG celecoxib 100mg x 2/day/month**	1,176 JPY (11.29 USD)	100mg x 2/day = 39.2 JPY (0.38 USD)
**Cost of pharmacotherapy for gastrointestinal bleeding/month**	2,787 JPY (26.76 USD)	92.9 JPY/day (0.89 USD/day) (weighted by branded/generic ratio in the national statistics 2017)
**Cost of prophylactic administration for gastrointestinal bleeding /month**	2,175 JPY (20.88 USD)	72.5 JPY/day (0.70 USD/day) (weighted by branded/generic ratio in the national statistics 2017)
**Subsequent visit fee**	730 JPY (7.01 USD)	Medical fee
**Dispensing fee**	2,080 JPY (19.97 USD)	Medical fee (basic dispensing fee: 42 points, standard dispensing addition 1:32 points, dispensing fee (oral dose): 77 points, medication history management instruction fee: 57 points)
**Acute treatment cost for gastrointestinal bleeding**		
**Hospitalization, pharmacotherapy**	200,360 JPY (1,923.46 USD)	DPC (hospital stay is assumed as day II)
**Hospitalization, operation**	292,950 JPY (2,812.32 USD)	DPC (hospital stay is assumed as day II) + endoscopic hemostasis
**Endoscopy cost**	11,400 JPY (109.44 USD)	Medical fee

AG: Authorized generic, DPC: Diagnosis procedure combination.

The cost for each drug was set as follows: 927 JPY/month (8.90 USD/month) based on loxoprofen tablets 60 mg (three times a day [180 mg dose]) as the weighted price of branded and generic loxoprofen 180 mg according to actual share in the national statistics 2017, and 4,140 JPY (39.74 USD) and 1,176 JPY (11.29 USD) per month based on branded and AG celecoxib tablets 100 mg (twice a day [200 mg dose]) as the price of celecoxib 200 mg, respectively.

The treatment cost for each event was set according to the medical fee and the score table of the Diagnosis Procedure Combination (DPC) system in Japan.

### Utility parameters

The utility parameters in the model were listed in Table 3.

**Table 3:** Utility parameters.

	Parameters	Setting	Source
**Utility value for underlying disease**	First line treatment	0.723	Utility value in arthritis patients taking NSAID
	Treatment of minor gastrointestinal injury	0.688	Utility value in arthritis patients without treatment
	Treatment of symptomatic ulcer	0.688	Utility value in arthritis patients without treatment
	Treatment of gastrointestinal bleeding	0.688	Utility value in arthritis patients without treatment
	Second line treatment after minor gastrointestinal injury	0.723	Utility value in arthritis patients taking NSAID
	Second line treatment after symptomatic ulcer	0.723	Utility value in arthritis patients taking NSAID
	Second line treatment of gastrointestinal bleeding	0.723	Utility value in arthritis patients taking NSAID
**Utility value for digestive tract injury**	Disutility in case of minor gastrointestinal injury	0	Quoting utility value 0.73 when indigestion occurs
	Disutility in case of symptomatic ulcer	-0.0144	(0.723–0.550) / 12 Assuming the symptom appearance period as 1 month, quoting the utility value of 0.55 for symptomatic ulcer.
	Disutility in case of gastrointestinal bleeding	-0.0219	(0.723–0.550) / 12 Assuming the symptom appearance period as 1 month, quoting the utility value of 0.55 for symptomatic ulcer.

QALY: Quality-adjusted life year, NSAIDs: Non-steroidal anti-inflammatory drugs.

In this analysis, the utility values were set based on the analysis by Latimer et al. (2009), in which a medical economic analysis of gastrointestinal injury caused by NSAIDs had been conducted, assuming UK patients [21]. The utility value represent relative QOL weight in the range of 0 (meaning death) to 1 (meaning full-health) scale to calculate quality-adjusted life year (QALY) for chronic pain with OA, RA, and LBP and those at the time of gastrointestinal injury.

In Latimer et al. (2009), the results of a meta-analysis looking at Western Ontario and McMaster Universities (WOMAC) scores were transformed into utility values by regression equations and set in the model. In this analysis, based on the setting of Latimer et al. (2009), the utility value of NSAIDs during first-line and second-line treatments was set to 0.723 [21].

The utility value after treatment period of each event and discontinuation of NSAIDs administration was set to decrease by 0.035 from the time of taking NSAIDs (0.688 as the utility value if NSAIDs are suspended). The analysis by Latimer et al. (2009) cited the reports of Maetzel, Krahn and Naglie (2002), which measured the utility value of each event for 60 Canadians by the Standard Gamble technique by looking at each event occurrence [22]. In this analysis and based on these settings, we assumed that the period (based on 1 month in the analysis) in which the utility value would decrease temporarily, and set the decrease of QALY due to the event occurrence: 0.0144 QALY in the case of symptomatic ulcer and 0.0219 QALY in the case of gastrointestinal bleeding.

### Sensitivity analysis

To examine the robustness of the analysis result, one-way and probabilistic sensitivity analyses were performed. For the sensitivity analysis range of values, a 95% confidence interval was employed for all probability and utility parameters, and +/-20% for the cost parameters. A probabilistic sensitivity analysis was performed with 1000 Monte-Carlo simulations to evaluate the uncertainty of the results. The stochastic and health state utility parameters were assumed to have a beta distribution, the disutility parameters to have a gamma distribution and the ratio parameters (e.g. relative ratio) to have a lognormal distribution. A scenario analysis with the lowest generic price of loxoprofen (i.e. 5.7 JPY (0.05 USD)) was also conducted as the most conservative analysis for AG celecoxib.

## Results

### Base-case and scenario analyses

The results of base-case analysis were shown in Table 4.

**Table 4:** Base-case analysis.

	Cost	Incremental Cost (vs. loxoprofen)	QALYs	Incremental QALYs (vs. loxoprofen)	ICER (vs. loxoprofen)
**Loxoprofen with weighted price by real-world branded/generic distribution**	1,003,910 JPY (9,637.54 USD)		13.741		
**Branded Celecoxib**	1,133,598 JPY (10,882.54 USD)	129,688 JPY (1,245.00 USD)	13.764	0.024	5,403,667 JPY/QALY (51,875.20 USD/QALY)
**AG Celecoxib**	997,642 JPY (9,577.36 USD)	-6,268 JPY (-60.17 USD)	13.764	0.024	Dominant

AG: Authorized generic, QALY: Quality-adjusted life years, ICER: Incremental cost-effectiveness ratio.

The cost-effectiveness of branded and AG celecoxib was evaluated and compared to loxoprofen with weighted price by conducting the Monte-Carlo simulations of the lifetime prognosis of the patients with chronic pain caused by OA, RA and LBP. As a result, celecoxib was expected to yield the incremental QALYs of 0.024 (in total, 13.764 in celecoxib vs.13.741 in loxoprofen).

On the other hand, the total costs for each strategy were 1,003,910 JPY (9,637.54 USD), 1,133,598 JPY (10,882.54 USD) and 997,642 JPY (9,577.36 USD) for loxoprofen with weighted price, branded celecoxib and AG celecoxib, respectively. Therefore, as a point estimate, a cost-increase of 129,688 JPY (1,245.00 USD) for branded celecoxib and a cost-reduction of 6,268 JPY (60.17 USD) for AG celecoxib were recognized per patient in lifetime horizon, compared to loxoprofen with weighted price. Hence, the ICER of branded celecoxib attained 5,403,667 JPY/QALY (51,875.20 USD/QALY), compared to loxoprofen with the weighted price.

**Fig 2:**
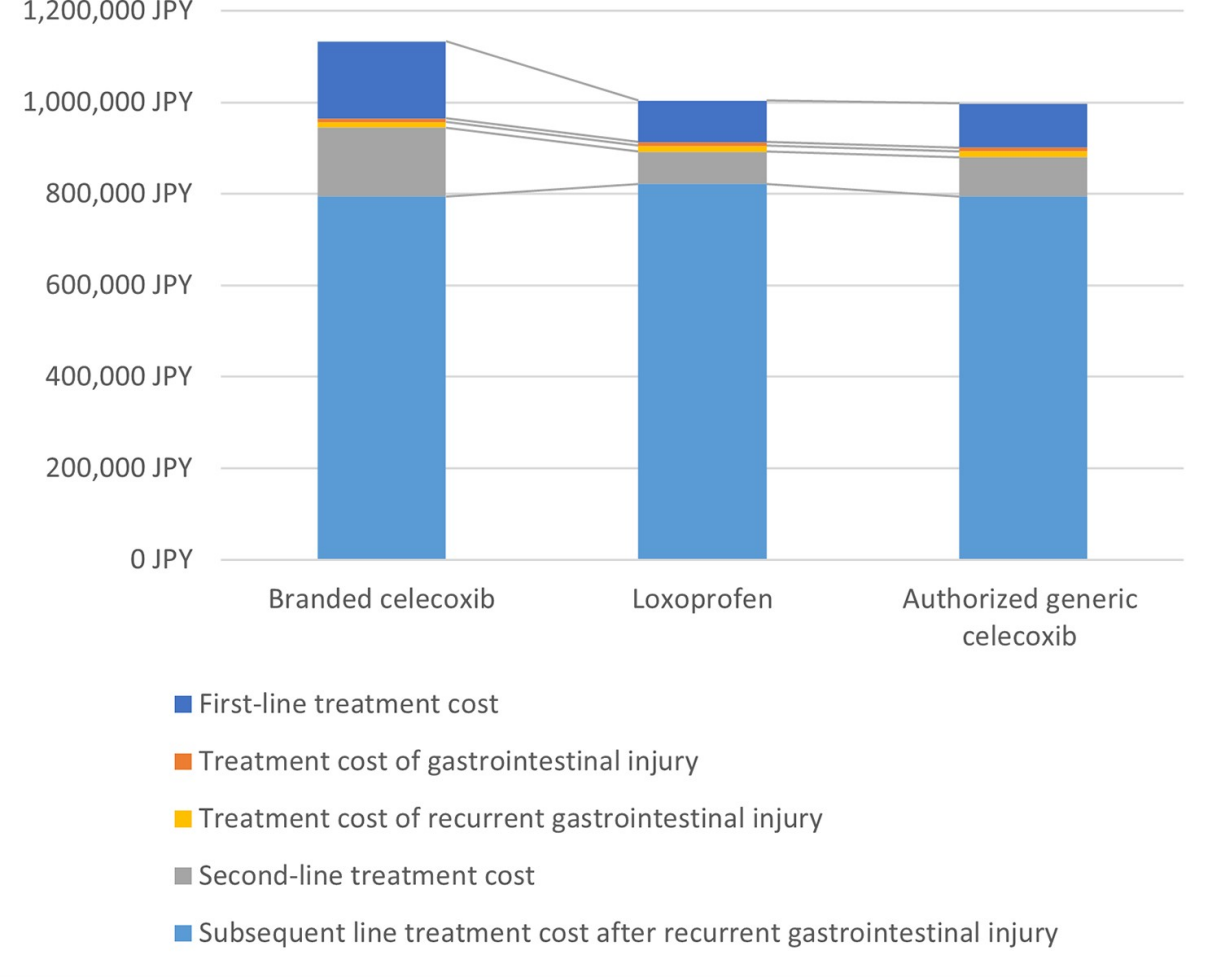
Lifetime cost breakdown for each strategy.

The breakdown of total cost for each strategy is shown in Fig 2. Although the costs of first-line treatment in celecoxib were increased against loxoprofen with weighted price, the costs including the treatment of events and subsequent treatment for recurrent prevention were significantly reduced leading to the balance of costs in total between two regimens.

The results of scenario analysis with the lowest generic price of loxoprofen are shown in Table 5. The total cost of AG celecoxib was slightly higher than that of generic loxoprofen and the ICER value of AG celecoxib was estimated at 417,583 JPY/QALY (4,008.80 USD/QALY).

**Table 5:** Scenario analysis with the lowest generic price of loxoprofen.

	Cost	Incremental Cost	QALYs	Incremental QALYs	ICER
**Generic Loxoprofen**	987,620 JPY (9,481.15 USD)		13.741		
**AG Celecoxib**	997,642 JPY (9,577.36 USD)	10,022 JPY (96.21 USD)	13.764	0.024	417,583 JPY/QALY (4,008.80 USD/QALY)

AG: Authorized generic, QALY: Quality-adjusted life years, ICER: Incremental cost-effectiveness ratio.

### Sensitivity analyses

**Fig 3:**
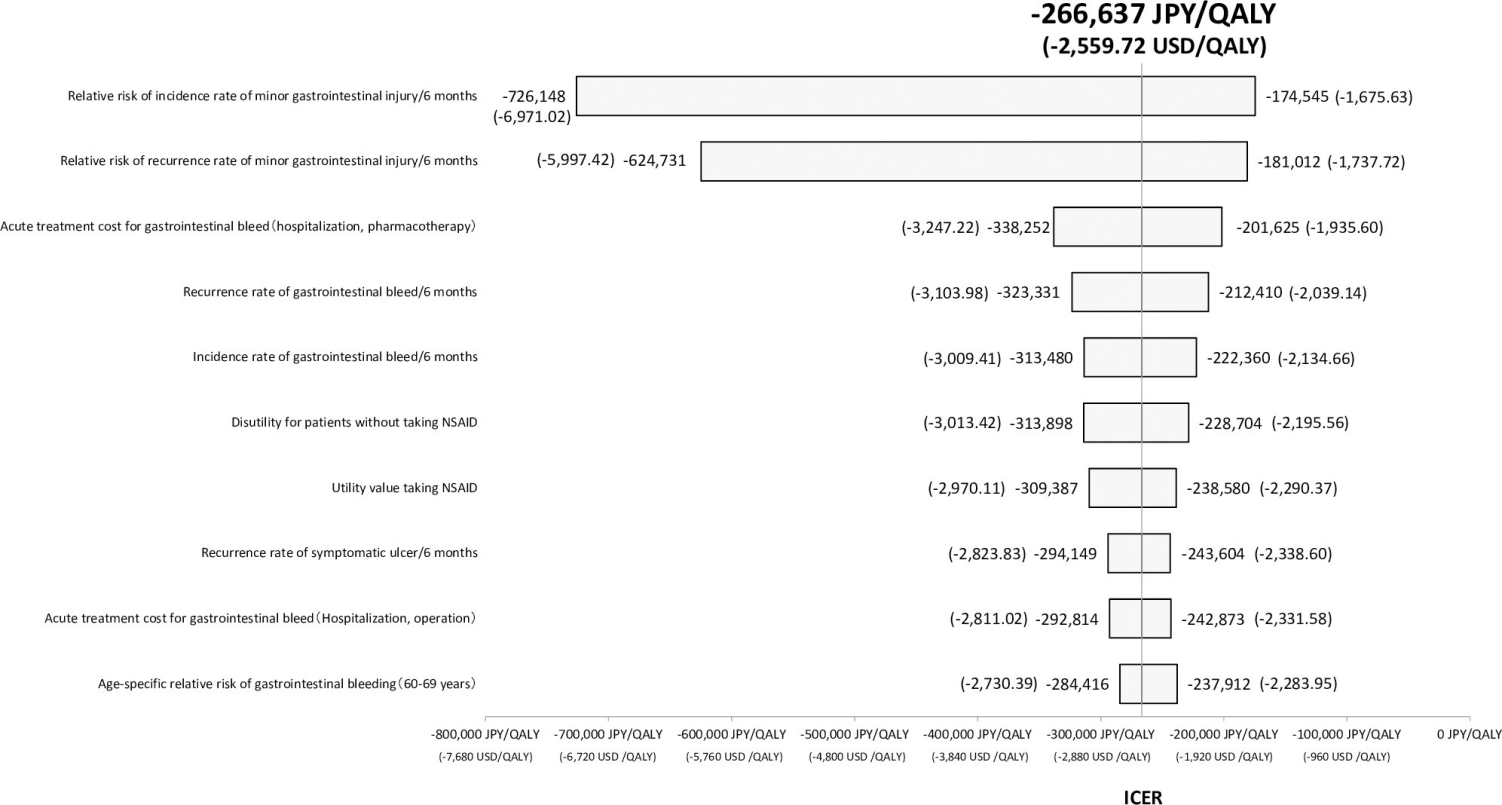
Tornado diagram of the 10 most sensitivity parameters in one-way sensitivity analyses. QALY, quality-adjusted life year; ICER, incremental cost-effectiveness ratio, NSAIDs: non-steroidal anti-inflammatory drugs.

Fig 3 illustrates the tornado diagram based on one-way sensitivity analysis with respect to the comparison of loxoprofen with weighted price vs. AG celecoxib. No case was recognized to reverse the results of cost-saving by AG celecoxib. If the most sensitive parameter for ICER value, the relative risk of incidence rate of minor gastrointestinal injury/6 months, was assumed to be the worst case (i.e. 1.36), the ICER calculation can be -174,545 JPY (-1,675.63 USD).

Each vertical bar represents the range that the ICER value can be take in one-way sensitivity analysis. The base case ICER value is shown as the central vertical line. The relative risk of minor gastrointestinal injury showed the greatest effect on the results of the analysis.

**Fig 4:**
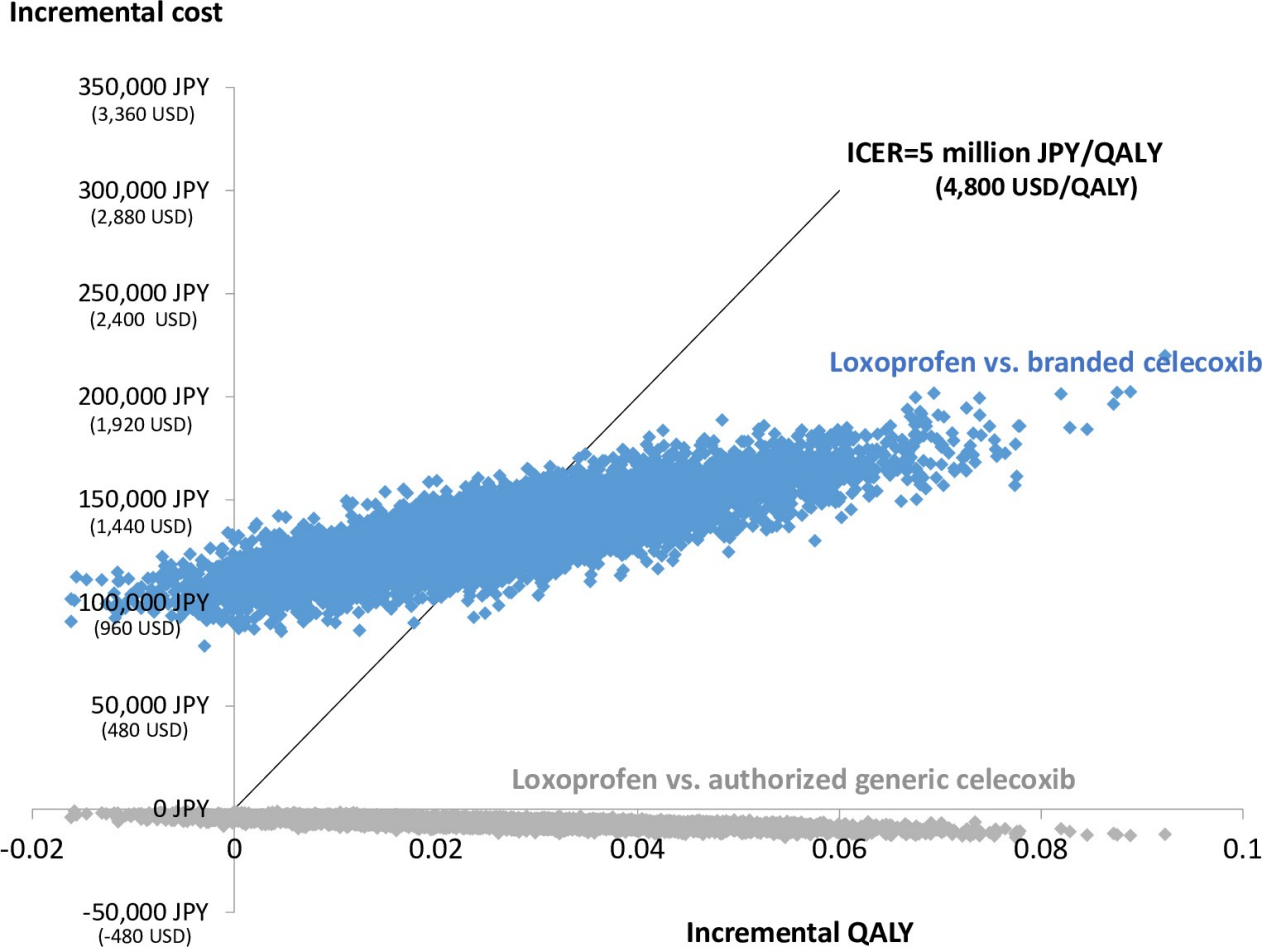
Scatter plot of pairs of incremental QALYs and costs. QALY, quality-adjusted life year; ICER, incremental cost-effectiveness ratio. The line indicates the reference value of the incremental cost-effectiveness ratio (ICER) (5 million JPY/quality-adjusted life year [QALY]) (48,000 USD/quality-adjusted life year [QALY]) in Japan. Probability of being cost-effective, percentage of plots located under the vertical line and the line of ICER value reference (5 million JPY/QALY) (48,000 USD/QALY) were 100% and 46.0%, respectively.

The results of probabilistic sensitivity analysis in the form of scattered plots for loxoprofen with weighted price vs. AG celecoxib and branded celecoxib are shown in Fig 4. The probability of cost-saving by AG celecoxib and being cost-effective in AG celecoxib or branded celecoxib against loxoprofen with weighted price (in considering a potential ICER threshold of 5 million JPY (48,000 USD)) were 100% and 46.0%, respectively, even if the uncertainties of each parameter were considered simultaneously.

## Discussion

Evaluation of AG celecoxib demonstrated that it would be dominant against loxoprofen with weighted price in a point estimate, although the amount of cost reduction was not substantial, and scenario analysis with the lowest generic price of loxoprofen showed the slightly higher lifetime cost in AG celecoxib compared with loxoprofen. Hence, the authors interpret the results of this analysis such that even if AG celecoxib is still slightly more expensive than generic loxoprofen, it effectively can improve the prognosis and quality of life of patients without meaningful cost impact.

Health technology assessment using cost-effectiveness analysis for price adjustment has been initiated from April 2019 in Japan. The ICER reference value for price reduction for usual cases is 5 million JPY (48,000 USD) and technologies are considered as cost-effective if the ICER value of those is below reference (if the ICER value of a new drug was exceeded the reference value then the price of that will be reduced by government) [23]. The ICER value of branded celecoxib was slightly higher than the reference value of 5 million JPY (48,000 USD) for price adjustment. Therefore, it would be reasonable to consider that branded celecoxib could be borderline cost-effective. The results of one-way sensitivity analysis and probabilistic sensitivity analysis showed the robustness of AG celecoxib and demonstrated that it would be dominant against loxoprofen with weighted price since there were no cases to range the first and second quadrant.

There was a difference in the ICER value between our analysis and the previous report (5,403,667 JPY/QALY vs. 5,001,167 JPY/QALY (51,875.20 USD/QALY vs. 48,011.20 USD/QALY)) in which the same model structure and concept were used because the settings of drug cost for loxoprofen and celecoxib were mainly higher and lower than this time, respectively (1,680JPY vs. 927JPY (16.13 USD vs. 8.90 USD) or loxoprofen, 3,640 JPY vs. 4,140 JPY (34.94 USD vs. 39.74 USD) for celecoxib) [9].

As a previous study on the cost-effectiveness of celecoxib, an analysis of OA patients was conducted by the National Institute for Health and Care Excellence (NICE) of the UK [24]. The analysis used a Markov model to consider gastrointestinal injuries caused by long-term administration of NSAIDs and considered the analysis period to be lifetime. Celecoxib was also evaluated to be cost-effective in the analysis by NICE.

Additionally, we also must consider several limitations for our interpretation. Firstly, we could not demonstrate an additional benefit of celecoxib on non-symptomatic gastrointestinal injury nor on disease prognosis. Non-symptomatic gastrointestinal injury would be relevant to the future symptomatic gastrointestinal event or recurrent risk and celecoxib could also reduce the risk of gastrointestinal injury including those that are non-symptomatic in nature, more strongly. Hence, the cost-effectiveness of celecoxib would be further improved than that estimated in our analysis if the benefit of celecoxib on non-symptomatic gastrointestinal event could be included in the next study. Second, the data availability was limited in the current study. No Japanese data were utilized for the utility parameters. Also, the data input for the parameters were limited only to the arthritis patients taking NSAIDs–although RA and LBP patients were not included. However, because of the one-way sensitivity analysis, we found those utility parameters to be less critical to the robustness of the results in the analysis and the negative impact of this limitation on the conclusion would not be substantial. Finally, most of the source evidence for the benefit of celecoxib against the comparator were reported about ten years ago. Although we had performed the literature review before conducting the present study, we could not find any updated information. We do not believe that the limitation would have a substantial impact on the conclusion, since there had not been any big change in standard of care for treating chronic pain patients using NSAIDs.

In conclusion, the current cost-effectiveness analysis for AG celecoxib revealed its good value for costs, considering the patients’ future risk of gastrointestinal injury; also, the impact on costs due to AG celecoxib against loxoprofen will be small. It implies that the disadvantage of AG celecoxib being slightly more expensive than generic loxoprofen could be offset by the good cost-effectiveness during the prognosis.
